# Efficient complement-mediated clearance of immunosuppressed T cells by macrophages

**DOI:** 10.3389/fimmu.2023.1183180

**Published:** 2023-05-16

**Authors:** Angela A. F. Gankema, Charita Furumaya, Sara Fernández-Hermira, Mark Hoogenboezem, Hanke L. Matlung, Robin van Bruggen, Taco W. Kuijpers

**Affiliations:** ^1^ Department of Molecular Hematology, Sanquin Research, Amsterdam University Medical Center (AUMC), University of Amsterdam, Amsterdam, Netherlands; ^2^ Department of Research Facilities, Sanquin Research, Amsterdam, Netherlands; ^3^ Department of Pediatric Immunology, Rheumatology and Infectious Diseases, Emma Children’s Hospital, Amsterdam University Medical Center (AUMC), University of Amsterdam, Amsterdam, Netherlands

**Keywords:** complement, iC3b, macrophages, MDSC activity, neutrophils, phagocytosis, T cells, trogocytosis

## Abstract

Cancer is one of the leading causes of death worldwide. Treatment outcome is largely dictated by the tumor type, disease stage, and treatment success rates, but also by the variation among patients in endogenous anti-tumor responses. Studies indicate that the presence of neutrophils in the tumor microenvironment is associated with a worse patient outcome due to their ability to suppress local anti-tumor T cell activity. Our previous studies investigated the mechanisms by which neutrophils suppress and damage T cells to become smaller in size (small T cells), debilitating their effector activities. Several studies indicate a role for tumor-associated macrophages in scavenging damaged or dead cells. We hypothesized that the observed lack of small T cells in the TME by confocal microscopy is due to immediate uptake by macrophages. In this study, we confirmed that indeed only the smaller, damaged T cells are taken up by macrophages, once serum-opsonized. Damaged T cells opsonized with complement factor C3 fragments were phagocytosed by macrophages, resulting in almost instantaneous and highly efficient uptake of these small T cells. Inhibition of the complement receptors CR1, CR3 and CR4 expressed by macrophages completely blocked phagocytosis. By contrast, actively proliferating T cells (large T cells) were neither impaired in neutrophil-MDSC activity nor opsonized for phagocytosis by macrophages. Rapid removal of damaged T cells suggests a role of complement and macrophages within the tumor microenvironment to clear suppressed T cells in cancer patients.

## Introduction

According to the World Health Organization (WHO), cancer is still one of the leading causes of death worldwide, despite the constant development of new therapies (1). In the last decades, research has mainly been focused on new immunotherapies, including checkpoint inhibitors directed to the programmed cell death protein 1 (PD-1)/programmed death-ligand 1 (PD-L1) and cytotoxic T-lymphocyte-associated protein 4 (CTLA-4) pathways (2). Overall, there is a high degree of variability among patient responses to these immunotherapies (3). Recently, an increasing number of studies have focused on the innate immune system and its role in tumor clearance. These studies show that different types of cancer patients have an increased percentage of circulating myeloid-derived suppressor cells (MDSCs) and that the presence of neutrophils is directly correlated with a worse patient outcome (4–6).

In humans, neutrophils are the most abundant leukocytes in the blood, with each day an estimate of 10^11^ cells emerging from the bone marrow and released into the bloodstream (7). Neutrophils have a half-life up to about 12 hours in the circulation, and are known to have a limited extravascular lifespan (7, 8). During invasion of bacteria or fungi, neutrophils participate in the innate immune response by killing the pathogen via phagocytosis, production of reactive oxygen species (ROS), degranulation, or formation of neutrophil extracellular traps (NETs) (9). All these processes can be activated by neutrophil-pathogen interactions via integrins present on their cell surface. In addition, neutrophils are also known to play a role in the adaptive immune system, especially during carcinogenesis. The tumor microenvironment (TME) can secrete CXC chemokine ligands, attracting neutrophils, which possess high levels of chemokine receptors CXCR1 and CXCR2, to the site of the tumor, in a process called chemotaxis (8). Additionally, inflammatory cytokines, such as TNFα, produced by the cancer or adjacent cells, lead to G-CSF production, prolonging neutrophil survival and stimulating granulopoiesis in the bone marrow, leading to more neutrophils in the TME (8). These neutrophils get activated and in their turn could secrete chemokines such as IL1β and IL-6, contributing to an inflammatory microenvironment observed in many solid tumors (6, 8, 10).

Neutrophils have been widely studied for their tumorigenic properties, mainly indirectly by suppressing the anti-tumorigenic activity of other immune cells (T cells). Upon stimulation, neutrophils exert various effector functions, such as ROS production by the NADPH oxidase complex, which converts oxygen radicals into hydrogen peroxide (H_2_O_2_) (11). H_2_O_2_ can suppress T cell proliferation in several ways, among which are ROS-induced damage (3, 12). Also degranulation plays a role in immunosuppressive activity (11). Three different types of granules can be identified; the azurophilic (primary) granules that contain myeloperoxidase (MPO), proteases, and defensins, specific (secondary) granules including lactoferrin, vitamin-B12-binding protein and the enzyme arginase-1, and gelatinase-containing (tertiary) granules (11–13). For instance, the release of arginase-1 leads to the conversion of L-arginine into urea and L-ornithine, inhibiting T cell proliferation and downregulating TCR expression (12). Both the production of ROS and secretion of MPO and other granule components, together with direct CD11b-dependent neutrophil-T cell interactions, are required for neutrophils to suppress T cell activity in a process called myeloid-derived suppressor cell (MDSC) activity (11, 14). It is thought that these processes contribute to a pro-tumorigenic environment since they prevent T cell activation and debilitate their effector activities to kill the tumor (11). We believe that the TME plays a role in defining the role of neutrophils, by secreting damage-associated molecular patterns (DAMPs), pro-inflammatory cytokines and chemokines, which could recruit and activate neutrophils to obtain an immunosuppressive phenotype (6).

As part of their suppressive activity on T cells, neutrophils can perform trogocytosis via CD11b/CD18-dependent interactions resulting in damaged, non-responsive “small” T cells (11). Small T cells are characterized by their altered morphology (forward scatter (FSC)^low^), a decrease in T cell activation markers and their severely energy-deprived metabolic state (11). As a consequence, they are no longer able to perform their anti-tumorigenic activities.

Since successful immunotherapy is still limited to a subgroup of cancer patients, better understanding of MDSC activity by neutrophils towards T cells in the TME may help to potentially overcome this cancer-induced immune evasion strategy by local neutrophils. With this in mind, an open question is what happens with small T cells when formed within the TME. Even though these small T cells are not undergoing apoptosis, we wondered whether the clearance process could be similar to efferocytosis, i.e. the uptake of apoptotic cells by professional (and non-professional) phagocytes (11). While different phagocytes are known to be involved in this process, in the context of the tumor microenvironment several studies have indicated a role for tumor-associated macrophages (TAMs) in scavenging damaged or dead cells as well as cellular debris in order to recycle cellular components (15–17). This process is essential for the maintenance of tissue homeostasis; defective efferocytosis may thus lead to a variety of chronic inflammatory diseases (16). We hypothesized that these generated small T cells can also be recognized and cleared by macrophages so that tissue homeostasis is maintained.

In this study, we generated small T cells by coculturing T cells with stimulated PMNs and analyzed the process of phagocytosis by macrophages. First we identified serum-opsonization to be essential for uptake of small T cells. Additionally, we investigated whether this process was antibody-mediated or dependent on the complement system and which complement receptors were involved in their uptake by macrophages.

## Method

### Isolation of immune cells

Heparinized peripheral blood was obtained from healthy donors, after giving informed consent, and diluted in a 1:1 ratio with phosphate-buffered saline (PBS) containing 10% (v/v) trisodium citrate (TSC). Immune cells were separated from each other by density gradient centrifugation over isotonic Percoll (Pharmacia, Uppsala, Sweden, 1.076 g/mL) at 800g at room temperature (RT). The interphase fraction, containing peripheral blood mononuclear cells (PBMCs), was collected for isolating T cells and/or monocytes. Cells were purified using magnetic-activated cell sorting (MACS) with either the Pan T cell isolation kit or the Pan monocyte isolation kit from Miltenyi-Biotec (Bergisch Gladbach, Germany) and used according to the manufacturer’s instructions. Polymorphonuclear cells (PMNs) were isolated from the pellet fraction after consecutive erythrocyte lysis by adding lysis buffer (155 mM NH_4_Cl, 10 mM KHCO_3_, 0.1 Mm EDTA) at 4°C. Subsequently, cells were resuspended in HEPES+ medium (containing 132 mM of NaCl, 20 mM HEPES, 6.0 mM KCl, 1.0 mM MgSO_4_, 1.0 mM CaCl_2_, 1.2 mM potassium phosphate, 5.5 mM glucose, and 0.5% (w/v) human serum albumin, pH 7.4). Cell concentrations were determined by the CASY Cell Counter (Roche, Basel, Switzerland).

### T cell fractionation after neutrophil-MDSC activity

To obtain small, damaged, T cells and larger proliferating human T cells, neutrophils and T cells from peripheral blood were isolated and co-cultured in a 1:1 ratio (0.1*10^6^ cells/well) in 96-well plates in medium containing IL-15 (0.1 ng/ml) and TNFα (10 ng/mL) for two days at 37°C. Before seeding, T cells were labelled with CellTracker™ Red (5 μM, Invitrogen, Thermo Scientific, Waltham, MA, USA) for 15 min at 37°C.

After two days, T cells were harvested and spun down at 300g for 10 min at 4°C, and resuspended in ice-cold FACS buffer (PBS containing 0.5% HSA). Cells were sorted with the BD FACSAria™ III cell sorter (BD Biosciences, San Jose, CA, USA) into small and large T cells according to forward/side scatter (FSC vs. SSC) (**Supplementary Figure 1B**) (11). The collected cell fractions were spun down and resuspended in HEPES+ medium, after which cells were divided over different test conditions.

### Phagocytosis assay

For the phagocytosis assay, monocytes were isolated (~95% purity) and cultured in IMDM-medium (Gibco, Life Technologies, Carlsbad, CA, USA) supplemented with 10% FCS (Bodinco, Alkmaar, The Netherlands), 10^4^ U/mL penicillin (Sigma-Aldrich), 10 ng/mL streptomycin (Sigma-Aldrich), 100 mM glutamine (Sigma-Aldrich, St. Louis, MO) and either granulocyte-macrophage colony-stimulating factor (GM-CSF) (10 ng/mL, PeproTech, Cranbury, NJ, USA) or macrophage colony-stimulating factor (M-CSF) (50 ng/mL, PeproTech). Monocytes were seeded at a density of 0.1*10^6^ cells per ibiTreated µ-Slide (Ibidi, Gräfelfing, Germany) for microscopy along with 0.5*10^5^ monocytes per well in 96-well flat-bottom plates for FACS and ImageStream. Half of the medium was refreshed on days 3 and 7 of culturing. At day 10, monocytes were polarized either towards M1 or M2 macrophages by adding GM-CSF or M-CSF, respectively, to the medium on day 0.

On day 10 following the neutrophil-MDSC activity culture, the purified T cell fractions (small and large T cells) were opsonized with 10% human serum (provided by Sanquin, pooled from 5 blood group AB positive healthy donors) for 20 minutes at 37°C. To study the role of the complement system in phagocytosis, serum was heat-inactivated for 1 hour at 56°C to inactivate complement activity. In some experimental conditions, the C3 inhibitor Cp40 or its scrambled peptide control (10 μM) (kindly provided by dr. Brahm Segal, Buffalo, NY) was added to the pooled serum prior to opsonization (18).

### Complement detection on macrophages and T cells

For cell surface expression analysis of macrophages and T cells, we used the Canto flow cytometer (BD Biosciences). Expression levels of CD11b-APC (clone D12, 1:150, BD Biosciences), CD11c-FITC (clone BU15, 1:10, Bio-Rad, Kidlington, UK), CD14-PECy7 (clone M5E2, 1:200, BD Biosciences), CD16-PECy7 (clone 3G8, 1:1000, BD Biosciences), CD18-FITC (clone MEM48, 1:50, Diaclone, Besançon cedex, France), CD32-FITC (clone AT10, 1:100, Bio-Rad), CD64-FITC (clone 10.1, 1:100, Bio-Rad), and CD163-APC (clone GHI/61, 1:50, Invitrogen) were measured on macrophages. CD35 (20 μg/mL, 1:500, kind gift from prof. dr. M.R. Daha, Leiden University Medical Center, The Netherlands) was used in combination with a secondary anti-rabbit antibody (AF633, 1:200, Invitrogen) on macrophages as well. T cells were stained with the following directly conjugated fluorescent antibodies: C3b-APC (clone 3E7/C3b, 1:100, BioLegend, San Diego, CA, USA) and C3d-FITC (clone BGRL 11, 1:100, ARP Products, Waltham, MA, USA) and primary antibodies C3-19 (Clone C3-19, 1:100, Sanquin, Amsterdam) and iC3b (1:20, Quidel, San Diego, CA), followed by the secondary anti-mouse antibody (α-ms-488, 1:200, Invitrogen).

### Blocking complement-mediated uptake of small T cells

Monoclonal CD11b blocking antibody 44a (10 μg/mL, isolated from the supernatant of hybridoma clones, obtained from the American Type Culture Collection, Rockville, MD), monoclonal CD18 blocking antibody IB4 (10 μg/mL, isolated from the supernatant of hybridoma clones, obtained from the American Type Culture Collection, Rockville, MD), monoclonal CD11c blocking antibody CBR-p150/4G1 (10 μg/mL, Invitrogen), and/or polyclonal CR1 blocking antibody (20 μg/mL, kind gift from prof. dr. M.R. Daha, Leiden University Medical Center, The Netherlands) were used as a single agent or in various combinations of receptor-blocking antibodies. Prior to the start of phagocytosis, macrophages were incubated with these blocking antibodies for 20 minutes at 37°C. Subsequently, T cells – either small or large, opsonized or non-opsonized – were added to macrophages in a 3:1 ratio (0.12*10^6^ T cells/well) and incubated for 1.5 hours at 37°C.

### Imaging of phagocytosis

Following the 1.5 hours macrophage-T cell coculture, the supernatant including remaining T cells was removed and macrophages were detached using a solution of 12.5 mM lidocaine/2mM EDTA. Cells were transferred to a V-bottom 96-well plate (Corning) by stringent pipetting for the different imaging methods.

For flow cytometry, the Fortessa flow cytometer (BD Biosciences) or LSR flow cytometer (BD Biosciences) was used for analyzing the phagocytosis data by FlowJo™ Software (v10.7.1, Tree Star, Inc, Ashland, OR, USA). After gating for the macrophages (based on the FSC vs SSC plot), the percentage of macrophages positive for CellTracker™ Red fluorescence of phagocytosed T cells was determined and quantified as percentage of positive cells. The geometric mean fluorescence intensity (gMFI) of macrophages was also determined.

For ImageStream, macrophages were labelled with CD18-FITC and analyzed with ImageStream Mark II (Merck) for Celltracker Red labelling (T cell-uptake) after phagocytosis as described above. Data were analyzed using the Software program IDEAS®.

For widefield microscopy we used the Nikon Eclipse Ti2 widefield microscope equipped with an Okolab CO_2_ unit at 37°C and a 20X air objective. To visualize the process of phagocytosis by live-cell imaging, T cells were added to macrophages in an Ibidi chamber and directly imaged. Videos were recorded for at least 2 hours with a time frame of 1 picture/min. NIS Elements software (ND acquisition) was used to record the time lapse, with excitation at 580 nm and TRITC (excitation 540/25, emission 605/55) bandpass filter cube. Videos were processed using ImageJ software (FIJI).

### Statistical analysis

Statistical tests were performed using GraphPad Prism 8 (San Diego, CA, USA). Data were analyzed with one-way ANOVA with a correction for multiple comparisons (Dunnett). Results are shown as means +/- standard error of the mean (SEM). P values <0.05 were considered statistically significant.

## Results

### Opsonized small T cells are phagocytosed by macrophages

Our previous *in vitro* work has shown that stimulated neutrophils can exert MDSC activity towards T cells, resulting in a population of small, damaged T cells, which are non-functional (11). Having stained solid tumor sections of various backgrounds with markers to determine the presence of T cells, we were as yet unable to detect any indication of such smaller T cells within the tumor microenvironment (6, and data not shown). This could either be due to a lack of resolution of the imaging machine used (AiryScan-2), the lack of trogocytosis by neutrophils towards T cells *in vivo*, or the rapid disappearance of such small T cells once generated. Since TAMs are known to scavenge damaged or dead cells and cellular debris within the TME, we investigated whether macrophages would recognize the immunosuppressed T cells *in vitro* (17).

To study the scavenging process, we set up a phagocytosis assay in which we culture macrophages, add damaged T cells (hereafter “small T cells”), generated by coculturing T cells and neutrophils, and/or the intact proliferating T cells (hereafter “large T cells”) (**Supplementary Figure 1A**). We found a maximum in the percentage of small T cells on day 2 of the coculture, after which we assume small T cells may be dying while large T cells start proliferating and we therefore observe a shift in the proportion small vs large T cells. Small and large T cells were sorted based on forward/sideward scatter, opsonized, and added to macrophages separately, after which we determined the percentage of Celltracker Red^+^ macrophages as a measure for phagocytosis (**Supplementary Figure 1C**).

**Figure 1:**
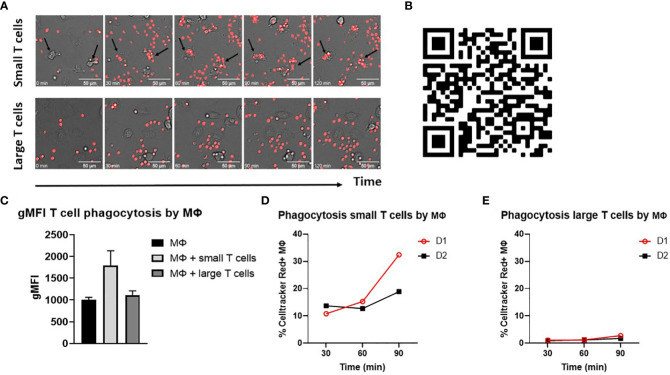
Opsonized small T cells are phagocytosed by macrophages. **(A)** Phagocytosis of opsonized small and large T cells (red) by M-CSF differentiated macrophages analyzed by widefield microscopy. Images are shown for five different time points starting from t=0 until t=120 min during phagocytosis. Arrows indicate examples of T cell uptake by macrophages. Scale bar is 50 μm. **(B)** Videos were created from images that were taken with a timeframe of 1 picture/min. **(C)** Geometric mean fluorescence intensity (gMFI) of macrophages only (background staining) or cocultured with opsonized small or large T cells for 120 min. **(D, E)** Time points (t=30, t=60, t=90 min) of small and large T cell phagocytosis and the percentage of Celltracker Red^+^ M-CSF macrophages of two different donors (D1 and D2). Small and large T cells were both opsonized with serum.

A clear distinction was observed between the phagocytosis of opsonized small T cells versus large T cells by widefield microscopy. Small T cells easily bound to and were phagocytosed by macrophages, while large T cells seemed to make some initial contact with macrophages but were not recognized, and thereby not phagocytosed (**Figures 1A, B**). When quantified by flow cytometry, the gMFI of macrophages cocultured with small T cells was also higher compared to macrophages cocultured with large T cells or macrophages without T cells as a control for background staining (**Figure 1C**). This confirmed the idea of phagocytosis of Celltracker Red-labelled small T cells, but not large T cells, by macrophages. In addition, a time-course experiment on phagocytosis of small and large T cells showed an increase in the phagocytosis of opsonized small T cells in time, while this increase was not found for large T cells, indicating that only small T cells were taken up by macrophages (**Figures 1D, E**). Based on this time course experiment together with microscopy data we decided to continue phagocytosis experiments for a time frame of 90 minutes, since this was the minimum amount of time needed to be able to make a clear distinction between small and large T cell uptake by macrophages. Furthermore, microscopy data had shown that after this period of time, phagocytosis slowed down (data not shown).

**Figure 2:**
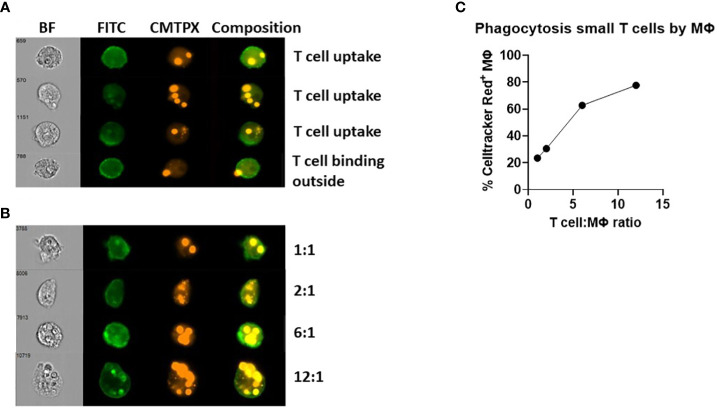
Phagocytosis of opsonized small T cells analyzed by imaging flow cytometry. **(A, B)** Macrophages were labelled with CD18-FITC and T cells were labelled with Celltracker Red (CMTPX). A composition of both channels was created to determine whether T cells were inside or outside the macrophage after 90 min of coculturing. **(B, C)** Different T cell:macrophage ratios (1:1 up to 12:1) were used to test the phagocytic capability of macrophages.

To prove that we measured T cell uptake by macrophages rather than sticking of T cells to the macrophages, we applied imaging flow cytometric analysis to distinguish T cells inside the macrophage from T cells solely binding to the cell membrane of macrophages. The composition of CD18-FITC labelled macrophages and Celltracker Red^+^ T cells clearly shows that opsonized small T cells were phagocytosed by macrophages (**Figure 2A**). Because large T cells were not phagocytosed by macrophages, we excluded them from further analysis (**Supplementary Figure 2**).

In this imaging flow cytometric analysis, we could also discriminate macrophages by the number of T cells phagocytosed and investigated different T cell:macrophage ratios (**Figures 2B, C**). These data suggested a correlation between the T cell:macrophage ratio and the percentage of Celltracker Red^+^ macrophages, reaching a plateau of about 80% of Celltracker Red^+^ macrophages at a 12:1 ratio. We decided to use a 3:1 ratio in most experiments as a minimum of cells needed to study small T cell uptake after 90 minutes, at the steep part of the curve, for optimal investigation of the effects of different serum treatment and blocking agents.

### Role of complement in T cell opsonization

**Figure 3:**
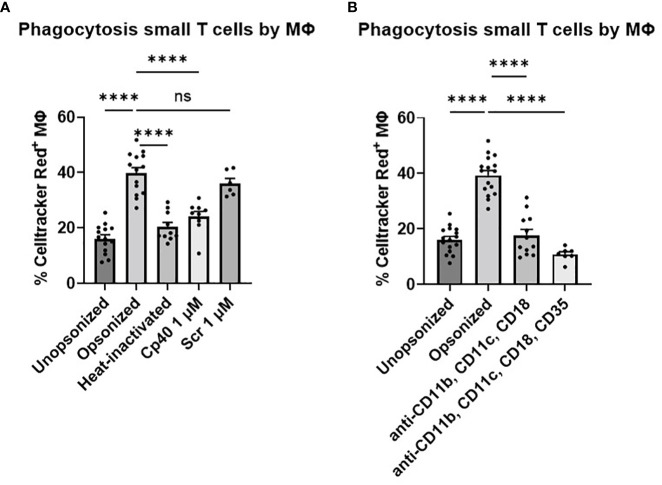
Phagocytosis of small T cells by M2 macrophages. **(A)** Small T cells either unopsonized or opsonized with serum, heat-inactivated serum, serum including complement inhibitor Cp40, or serum including a scrambled peptide control (Scr) cocultured with M-CSF macrophages. **(B)** Opsonized small T cells in combination with blockage of macrophage receptors; blocking MoAbs 44a against CD11b, CBR-p150/4G1 against CD11c, IB4 against CD18, and CR1 against CD35 cocultured with M-CSF macrophages. Each dot represents data from a single donor. Shown are P-values <0.05. ****P<0.001, ns = not significant, n, 4-11.

Since we found that only opsonized small T cells were phagocytosed by macrophages, this process could either be antibody-mediated or regulated by the complement system (**Figure 3A**). To determine this, we included a condition in which we heat-inactivated the serum for 1 hour at 56°C, inactivating all complement factors while the antibodies remained intact. When we compared this condition to the opsonized condition, we observed a decrease in phagocytosis (P<0.0001), indicating an important role for complement in the phagocytosis of small T cells (**Figure 3A**). To further confirm that complement activation is essential for subsequent phagocytosis, we used the complement inhibitor Cp40, which works by interfering with convertase formation and C3 cleavage (18). Opsonization of small T cells with serum containing Cp40 also resulted in a decrease in phagocytosis compared to its scrambled peptide control (P<0.001), contributing to the idea that complement plays a major role in marking cells for phagocytosis.

**Figure 4:**
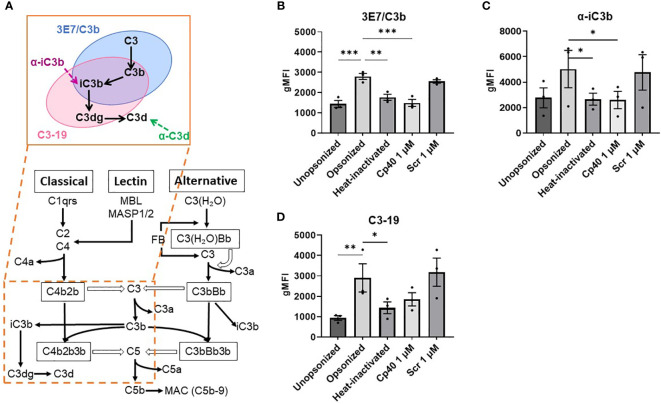
Binding of iC3b, C3-19, and 3E7/C3b antibodies to opsonized small T cells is reduced after inhibition or inactivation of complement factors in the serum. **(A)** Simplified diagram of the complement cascade and binding of the antibodies used. **(B–D)** Graphs show the geometric mean fluorescence intensity (gMFI) measured by flow cytometry of the antibodies binding to different conditions of small T cells: unopsonized, opsonized with serum, opsonized with heat-inactivated serum, opsonized with Cp40-treated serum, opsonized with scrambled peptide control. All conditions are compared to the opsonized condition. Shown are P-values<0.05. *P<0.05, **P<0.01, ***P<0.005, n = 3.

Finally, opsonized small T cells showed higher levels of complement factor surface staining in comparison with the unopsonized condition (**Figures 4B–D**). When complement proteins were denatured by heat inactivation, or C3 cleavage was blocked with the Cp40 inhibitor, the levels of bound complement factors decreased significantly and were comparable to the unopsonized condition, while the scrambled peptide control showed levels similar to opsonized cells. Different antibodies were used to detect different complement factors present on the T cell surface (**Figure 4A**). Although uncertain about the exact epitope specificity on C3 fragments, MoAb 3E7/C3b was used to detect C3, C3b and iC3b, MoAb a-iC3b for specifically detecting iC3b, MoAb C3-19 has been described to detect C3b, iC3b and C3dg, and MoAb BGRL 11/C3d was used for recognizing C3d. For the C3d antibody we were not able to observe fluorescent staining above background staining. We also evaluated expression levels of complement activation and deposition on large T cells, but those values were negligible compared to small T cells (**Supplementary Figure 3**).

### CR3 and CR4 recognize iC3b expression on opsonized small T cells

Being interested in which receptors on the cell membrane of macrophages were involved in the recognition of the complement factors deposited on the membrane of small T cells, we blocked the complement receptors CR1 (CD35), CR3 (CD11b/CD18) and CR4 (CD11c/CD18) and studied the effect on phagocytosis of the small T cells. Only blocking the β2 integrin receptor CD11b/CD18 or CD11c/CD18 did not lead to a complete decrease in phagocytosis, but blocking both receptors did (P<0.001) (**Figure 3B**; **Supplementary Figure 4**). Furthermore, blocking CR1 in addition to CR3 and CR4 seemed to lower the phagocytosis slightly more (but only when combined with the other blocking antibodies), emphasizing the involvement of (i)C3b in this process.

Furthermore, we checked for CD47 expression levels on both small and large T cells either unopsonized, opsonized with serum or opsonized with heat-inactivated serum but no significant differences could be observed between the different conditions (data not shown), meaning that the CD47/SIRP interaction seemed not to be involved in the macrophages uptake of small T cells.

### Both M1 and M2 macrophages are capable of phagocytosing opsonized small T cells

We mainly studied phagocytosis by M-CSF induced macrophages since most TAMs within the TME are of the M2 type. TAMs are associated with tumor growth, immunosuppressive function and efferocytosis of apoptotic cells (15, 19, 20). We also studied the phagocytic capacity of GM-CSF induced M1 macrophages in parallel, which are more associated with infections and inflammation.

Successful polarization towards M1 or M2 macrophages was determined by measuring FcγRI, FcγRII, FcγRIII and CD163 surface expression levels by FACS (**Supplementary Figure 5A**) and we compared the CR1, CR3 and CR4 expression levels, as well as their phagocytic activity (**Supplementary Figures 5B, C**) (21). All macrophages expressed CR1, CR3, and CR4, irrespective of their M1 or M2 phenotype. The gMFI for CD11b was expressed in M2 macrophages at a slightly lower level (**Supplementary Figure 5B**). When comparing the phagocytic capacity of GM-CSF and M-CSF differentiated macrophages, we also did not observe significant differences between M1 and M2 macrophages (**Supplementary Figure 5C**). In line with these findings, we observed a similar phagocytic response of both types of macrophages with serum-opsonized small T cells, which – again – was almost completely reduced when using heat-inactivated serum lacking active complement factors. This indicates that independent on the type of macrophages opsonized small T cells can be rapidly phagocytosed.

## Discussion

Neutrophils are able to suppress T cell proliferation, facilitated by several toxic mechanisms including trogocytosis, leading to small, damaged and non-responsive T cells, as we had previously demonstrated in our *in vitro* studies (6, 11). Since there is no specific marker known for small T cells we have tried to discriminate them based on size but could not find any smaller T cell (fragments) in the histology section, suggesting these small T cell are not detectable within the TME with the techniques applied (6). For this reason, we focused on the possibility of a very rapid disappearance and clearance of small T cells. Because of the numerous TAMs within the TME of solid tumors (17), we assumed these cells could be held responsible for the absence of small T cells *in vivo*. In support of this assumption, we identified macrophages to clear small T cells by phagocytosis almost immediately, but only when opsonized with C3 fragment. Heat-inactivation of serum or usage of serum together with a complement inhibitor led to a decrease in phagocytosis comparable to the unopsonized condition. Flow cytometry analysis of small T cells revealed that mainly the deposition of complement proteins (i)C3b on small T cells are relevant for their uptake by macrophages. We found a significant increase in (i)C3b labelling on the cell surface of small T cells opsonized with serum compared to unopsonized cells or cells either opsonized with complement-inactivated serum or serum containing the small-compound Cp40 to block complement activation at the level of C3 (18). Since three different antibodies (MoAbs 3E7/C3b, a-iC3b and C3-19) are believed to recognize inactivated (i)C3b and staining following opsonization seems to be comparable, our findings may favor the idea of C3b being the most relevant complement fragment involved in small T cell phagocytosis by macrophages. In contrast to CR3 (CD11b/CD18), CR4 (CD11c/CD18) has been reported to recognize iC3b as well as C3dg (22, 23). However, the role of C3dg as an opsonin remains uncertain (24). We did not see a stronger blocking effect of anti-CD11c/CD18 versus anti-CD11b/CD18. Most likely, CR3 and CR4 cooperate as complement receptors on macrophages in complement-dependent phagocytosis, being only reduced significantly when both CR3 and CR4 are blocked. Furthermore, blocking CR1, which is believed to recognize C3b, C4b and iC3b, adds up to inhibit phagocytosis completely (25). Given that iC3b can bind to all three receptors, inhibition of only one of these complement receptors is inefficient for blocking small T cell phagocytosis (26).

Several studies show the presence of complement factors in tumor tissue (27). Complement factors may be produced and released by macrophages as well as tumor cells, which could explain the supposed absence of small T cells within the TME because of macrophage uptake (28–30). Plasma leakage could alternatively explain complement activity within the TME as a result of tumor neoangiogenesis or local factors inducing an increased vascular permeability (31, 32). With the lack of antibody mediated uptake, we may suggest that there is no role for a specific IgG binding to small T cells. Although IgM binding to damaged small T cells has not been formally excluded, a role for classical complement deposition seems with our findings less likely to occur. This would leave the alternative complement activation (and possibly the lectin pathway) the most likely route of opsonization. To further verify this we can take advantage of complement component-depleted human serum, for instance C4-depleted or factor D-depleted serum, for small T cell phagocytosis.

A role for complement in the TME was indicated before (6, 33). This was also demonstrated by neutrophils activated with ascites from patients with ovarian cancer causing complement-dependent suppression of tumor-associated T cells (26). Our results support a role for the complement system in the rapid clearance of small T cells by macrophages. Only small T cells opsonized with serum were phagocytosed by macrophages, which could be blocked by complement inactivation or inhibition by the Cp40 complement inhibitor, making the nonspecific binding of antibodies or other soluble proteins to the damaged T cells less likely to contribute to the phagocytic clearance of these damaged T cells.

Together with our previous findings on *in vitro* neutrophil-MDSC activity, we hypothesized that the process of trogocytosis by neutrophils, nibbling off pieces of T cell membrane, would also happen *in vivo* within the TME. Especially since we have proved the presence of PMNs, macrophages and CD8^+^ T cells in a histology section of colon carcinoma, which also showed clear contact between PMN and T cells (6). What is also important to notice is that trogocytosis does occur in case of ovarium carcinoma (26). However, to date we lack a definite marker to identify human neutrophil-MDSCs apart from their functional suppressive activity (6). Indeed, neutrophil-MDSC activity was shown to be induced in ascites and pleura exudates of cancer patients (26). Although we cannot assess the *in vivo* presence of small T cells in the TME of solid tumors by immunohistochemistry, our *in vitro* study suggests that neutrophil MDSC activity *in vivo* may be followed by uptake of suppressed T cells by local TAMs. In addition, an important role for the complement system in clearing small T cells has now been demonstrated *in vitro*.

## Data availability statement

The raw data supporting the conclusions of this article will be made available by the authors, without undue reservation.

## Author contributions

TK, AG, and CF conceived and designed the study. AG, CF, SF-H, and MH performed the experiments. HM and RB contributed to the design of the study. TK and AG wrote the manuscript. CF, HM and RB provided critical input and corrected the manuscript. All authors contributed to the article and approved the submitted version.

## References

[1] Sung H Ferlay J Siegel RL Laversanne M Soerjomataram I Jemal A. Global cancer statistics 2020: GLOBOCAN estimates of incidence and mortality worldwide for 36 cancers in 185 countries. CA Cancer J Clin (2021) 71(3):209–49. doi: 10.3322/caac.21660 33538338

[2] Sharma P Allison JP. The future of immune checkpoint therapy. Science (2015) 348(6230):56–61. doi: 10.1126/science.aaa8172 25838373

[3] Furumaya C Martinez-Sanz P Bouti P Kuijpers TW Matlung HL. Plasticity in pro- and anti-tumor activity of neutrophils: shifting the balance. Front Immunol (2020) 11(2100). doi: 10.3389/fimmu.2020.02100 PMC7492657 32983165

[4] Solito S Marigo I Pinton L Damuzzo V Mandruzzato S Bronte V. Myeloid-derived suppressor cell heterogeneity in human cancers. Ann N Y Acad Sci (2014) 1319(1):47–65. doi: 10.1111/nyas.12469 24965257

[5] Diaz-Montero CM Salem ML Nishimura MI Garrett-Mayer E Cole DJ Montero AJ. Increased circulating myeloid-derived suppressor cells correlate with clinical cancer stage, metastatic tumor burden, and doxorubicin-cyclophosphamide chemotherapy. Cancer Immunol Immunother (2009) 58(1):49–59. doi: 10.1007/s00262-008-0523-4 18446337 PMC3401888

[6] Segal B Giridharan T Suzuki S Khan A Zsiros E Emmons T. Neutrophil interactions with T cells, platelets, endothelial cells, and of course tumor cells. Immunol Rev (2022) 314:13–35. doi: 10.1111/imr.13178 PMC10174640 36527200

[7] Bouti P Webbers SDS Fagerholm SC Alon R Moser M Matlung HL. β2 integrin signaling cascade in neutrophils: more than a single function. Front Immunol (2021) 11. doi: 10.3389/fimmu.2020.619925 PMC7930317 33679708

[8] Jaillon S Ponzetta A Di Mitri D Santoni A Bonecchi R Mantovani A. Neutrophil diversity and plasticity in tumour progression and therapy. Nat Rev Cancer (2020) 20(9):485–503. doi: 10.1038/s41568-020-0281-y 32694624

[9] Liew PX Kubes P. The neutrophil’s role during health and disease. Physiol Rev (2019) 99:1223–48. doi: 10.1152/physrev.00012.2018 30758246

[10] Mussbacher M Salzmann M Brostjan C Hoesel B Schoergenhofer C Datler H. Cell type specific roles of nf-kb linking inflamation and thrombosis. Front Immunol (2019) 10(FEB):1–31. doi: 10.3389/fimmu.2019.00085 30778349 PMC6369217

[11] Aarts CEM Hiemstra IH Béguin EP Hoogendijk AJ Bouchmal S van Houdt M. Activated neutrophils exert myeloid-derived suppressor cell activity damaging T cells beyond repair. Blood Adv (2019) 3(22):3562–74. doi: 10.1182/bloodadvances.2019031609 PMC6880908 31738831

[12] Aarts CEM Kuijpers TW. Neutrophils as myeloid-derived suppressor cells. Eur J Clin Invest (2018) 48:e12989. doi: 10.1111/eci.12989 29956819

[13] Sheshachalam A Srivastava N Mitchell T Lacy P Eitzen G. Granule protein processing and regulated secretion in neutrophils. Front Immunol (2014) 5(448). doi: 10.3389/fimmu.2014.00448 PMC4168738 25285096

[14] Ohl K Tenbrock K. Reactive oxygen species as regulators of MDSC-mediated immune suppression. Front Immunol (2018) 9(2499):1–7. doi: 10.3389/fimmu.2018.02499 30425715 PMC6218613

[15] Boada-Romero E Martinez J Heckmann BL Green DR. The clearance of dead cells by efferocytosis. Nat Rev Mol Cell Biol (2020) 21:398–414. doi: 10.1038/s41580-020-0232-1 32251387 PMC7392086

[16] Doran AC Yurdagul A Tabas I. Efferocytosis in health and disease. Nat Rev Immunol (2020) 20(4):254–67. doi: 10.1038/s41577-019-0240-6 PMC7667664 31822793

[17] Myers KV Amend SR Pienta KJ. Targeting Tyro3, axl and MerTK (TAM receptors): implications for macrophages in the tumor microenvironment. Mol Cancer (2019) 18(94). doi: 10.1186/s12943-019-1022-2 PMC6515593 31088471

[18] Reis ES DeAngelis RA Chen H Resuello RRG Ricklin D LJ. Therapeutic C3 inhibitor Cp40 abrogates complement activation induced by modern hemodialysis filters. Physiol Behav (2015) 220(4):476–82. doi: 10.1016/j.imbio.2014.10.026 PMC4355228 25468722

[19] Ghate A Sharma S Agrawal P Sahu A. Differential expression of complement receptors CR1/2 and CR4 by murine M1 and M2 macrophages. Mol Immunol (2021) 137:75–83. doi: 10.1016/j.molimm.2021.06.003 34229135

[20] Mantovani A Allavena P Marchesi F Garlanda C. Macrophages as tools and targets in cancer therapy. Nat Rev Drug Discov (2022) 21(11):799–820. doi: 10.1038/s41573-022-00520-5 35974096 PMC9380983

[21] Nagelkerke SQ Bruggeman CW den Haan JMM Mul EPJ van den Berg TK van Bruggen R. Red pulp macrophages in the human spleen are a distinct cell population with a unique expression of fc- g receptors. Blood Adv (2018) 2(8):941–53. doi: 10.1182/bloodadvances.2017015008 PMC5916003 29692344

[22] van Lookeren Campagne M Wiesmann C Brown EJ. Macrophage complement receptors and pathogen clearance. Cell Microbiol (2007) 9(9):2095–102. doi: 10.1111/j.1462-5822.2007.00981.x 17590164

[23] Shinjyo N Kagaya W Pekna M. Interaction between the complement system and infectious agents – a potential mechanistic link to neurodegeneration and dementia. Front Cell Neurosci (2021) 15. doi: 10.3389/fncel.2021.710390 PMC8365172 34408631

[24] Vandendriessche S Cambier S Proost P Marques PE. Complement receptors and their role in leukocyte recruitment and phagocytosis. Front Cell Dev Biol (2021) 9(February):1–25. doi: 10.3389/fcell.2021.624025 PMC7905230 33644062

[25] Ricklin D Hajishengallis G Yang K LJ. Complement: a key system for immune surveillance and homeostasis. Nat Immunol (2010) 11(9):785–97. doi: 10.1038/ni.1923 PMC2924908 20720586

[26] Emmons TR Giridharan T Singel KL Khan ANMNH Ricciuti J Howard K. Mechanisms driving neutrophil-induced t-cell immunoparalysis in ovarian cancer. Cancer Immunol Res (2021) 9(7):790–810. doi: 10.1158/2326-6066.CIR-20-0922 33990375 PMC8287091

[27] Roumenina LT Daugan MV Petitprez F Sautès-Fridman C Fridman WH. Context-dependent roles of complement in cancer. Nat Rev Cancer (2019) 19:698–715. doi: 10.1038/s41568-019-0210-0 31666715

[28] Zhang R Liu Q Li T Liao Q Zhao Y. Role of the complement system in the tumor microenvironment. Cancer Cell Int (2019) 19(300):1–12. doi: 10.1186/s12935-019-1027-3 31787848 PMC6858723

[29] Singel KL Emmons TR Khan ANH Mayor PC Shen S Wong JT. Mature neutrophils suppress T cell immunity in ovarian cancer microenvironment. JCI Insight (2019) 4(5):e122311. doi: 10.1172/jci.insight.122311 30730851 PMC6483507

[30] Cho M Vasquez H Rupaimoole R Pradeep S Wu S Zand B. Autocrine effects of tumor-derived complement. Cell Rep (2014) 6(6):1085–95. doi: 10.1016/j.celrep.2014.02.014 PMC4084868 24613353

[31] Lugano R Ramachandran M Dimberg A. Tumor angiogenesis: causes, consequences, challenges and opportunities. Cell Mol Life Sci (2020) 77:1745–70. doi: 10.1007/s00018-019-03351-7 PMC7190605 31690961

[32] Cai A Chatziantoniou C Calmont A. Vascular permeability: regulation pathways and role in kidney diseases. Nephron (2021) 145(3):297–310. doi: 10.1159/000514314 33744890

[33] Ajona D Pajares MJ Corrales L Perez-Gracia JL Agorreta J Lozano MD. Investigation of complement activation product C4d as a diagnostic and prognostic biomarker for lung cancer. J Natl Cancer Inst (2013) 105(18):1385–93. doi: 10.1093/jnci/djt205 PMC3776260 23940286

