# Supplementary material for: Efficient complement-mediated clearance of immunosuppressed T cells by macrophages
Source: Front Immunol. 2023 May 16;14:1183180. doi: 10.3389/fimmu.2023.1183180 (PMC10228723; doi:10.3389/fimmu.2023.1183180)
Supplement: Supplementary file 1 [file DataSheet_1.docx]

***Supplementary Material***

**Efficient complement-mediated clearance of immunosuppressed T cells by macrophages**

Angela A.F. Gankema*^1^, Charita Furumaya^1^, Sara Fernández-Hermira^1^, Mark Hoogenboezem^2^, Hanke L. Matlung^1^, Robin van Bruggen^1^, Taco W. Kuijpers^1 3^

***Correspondence:** Angela A.F. Gankema: [a.gankema@sanquin.nl](mailto:a.gankema@sanquin.nl)


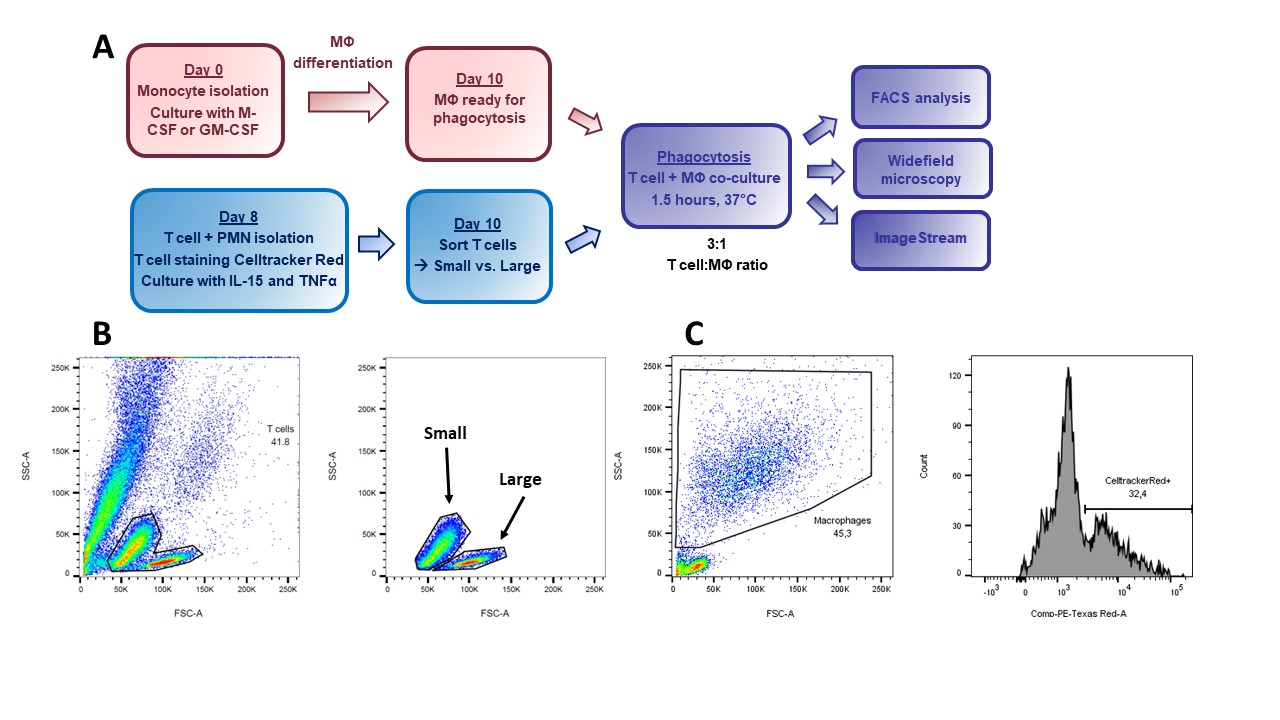


**Supplementary Figure 1. Setup of phagocytosis assay. A** Schematic overview phagocytosis assay. **B** Forward- and sideward scatter (FSC/SSC) plot showing the gating strategy to distinguish small T cells from large T cells on day 2 of T cell & neutrophil coculturing. **C** Gating strategy for determining the percentage of macrophages that became Celltracker Red^+^, meaning that they phagocytosed at least one T cell, as measured by FACS.


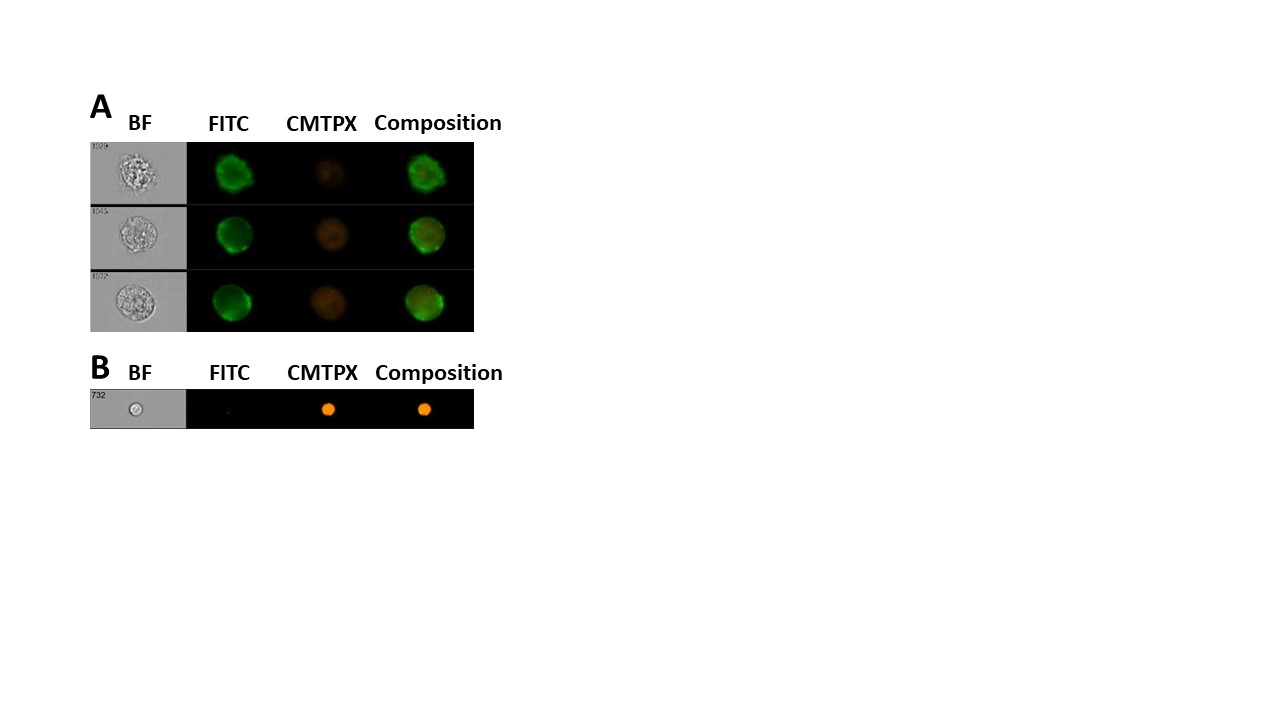


**Supplementary Figure 2. Lack of phagocytosis of opsonized large T cells as analyzed by imaging flow cytometry. A, B** Macrophages were labelled with CD18-FITC and T cells were labelled with Celltracker Red (CMTPX). A composition of both channels was created to determine whether T cells were taken up by macrophages after 90 min of coculturing. **A** Opsonized large T cells are not found inside macrophages, while **B** large T cells do show Celltracker Red labelling.

**
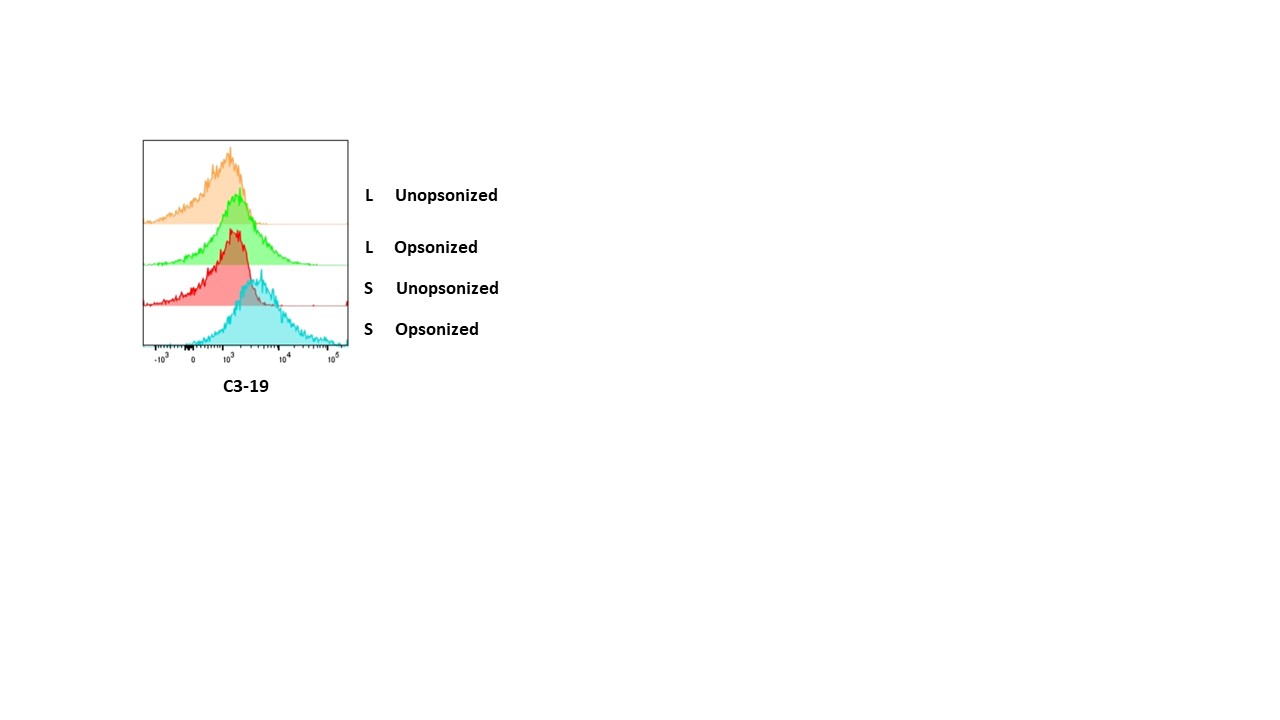
**

**Supplementary Figure 3. Opsonized large T cells show less C3-19 deposition compared to opsonized small T cells. A** C3-19 antibody deposition on both small (S) and large (L) T cells either unopsonized or opsonized with serum.


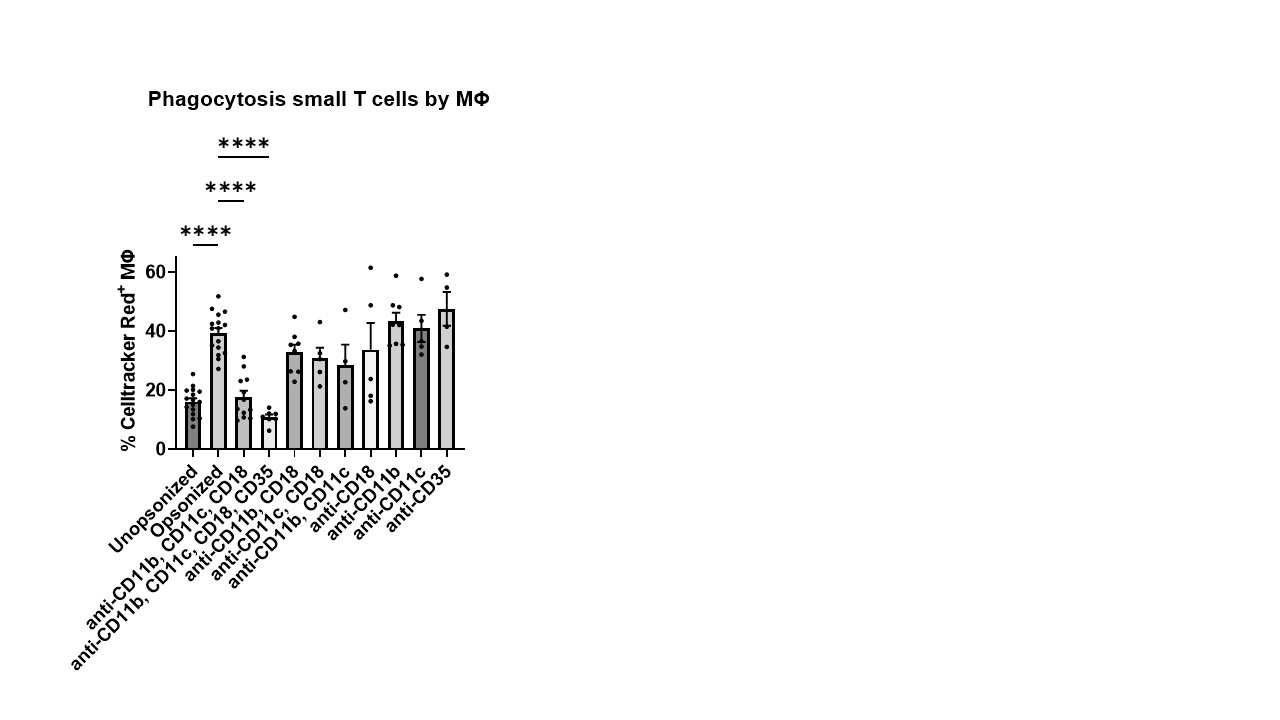


**Supplementary Figure 4. Blocking CR1, CR3, and CR4 leads to a decrease in phagocytosis of opsonized small T cells.** Phagocytosis of opsonized small T cells compared to several conditions in which (part of) macrophage receptors are blocked by different antibodies. 44a was used for blocking CD11b, IB4 for CD18, CBR-p150/4G1 for CD11c and CR1 for CD35. All conditions are compared to the opsonized condition. Only significant differences are shown. ****P<0.001, n = 4-11.


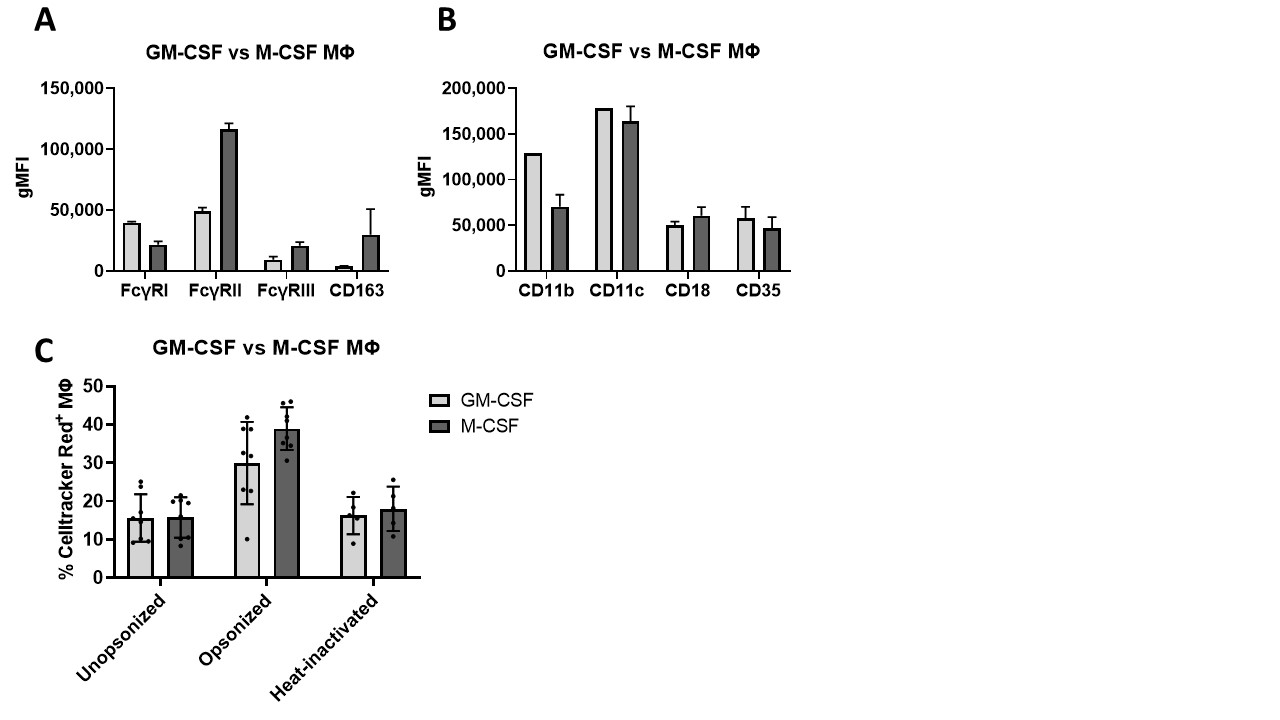


**Supplementary Figure 5. Differences between GM-CSF and M-CSF differentiated macrophages. A** FACS panel including FcγRI, FcγRII, FcγRIII, and CD163, and **B** CD11b, CD11c, CD18 and CD35 for M1 and M2 macrophages characterization, shown are the gMFIs based on the whole macrophage population, differences were not significant. **C** Phagocytosis of small T cells by GM-CSF (left bar) or M-CSF (right bar) differentiated macrophages. Small T cells were either unopsonized or opsonized with serum or heat-inactivated serum.
